# The Role of Microglia in Glioblastoma

**DOI:** 10.3389/fonc.2020.603495

**Published:** 2021-01-29

**Authors:** Noelia Geribaldi-Doldán, Cecilia Fernández-Ponce, Roberto Navarro Quiroz, Ismael Sánchez-Gomar, Lorena Gómez Escorcia, Erika Puentes Velásquez, Elkin Navarro Quiroz

**Affiliations:** ^1^ Departamento de Anatomía y Embriología Humanas, Facultad de Medicina, Universidad de Cádiz, Cádiz, Spain; ^2^ Instituto de Investigación e Innovación Biomédica de Cádiz (INiBICA), Cádiz, Spain; ^3^ Departamento de Biomedicina, Biotecnología y Salud Pública. Facultad de Medicina, Universidad de Cádiz, Cádiz, Spain; ^4^ CMCC-Centro de Matemática, Computação e Cognição, Laboratório do Biologia Computacional e Bioinformática–LBCB, Universidade Federal do ABC, Sao Paulo, Brazil; ^5^ Faculty of Basic and Biomedical Sciences, Universidad Simón Bolívar, Barranquilla, Colombia; ^6^ Centro de investigación e innovación en Biomoleculas, Care4You, Barranquilla, Colombia

**Keywords:** glioblastoma, microglia, signaling pathways, therapeutic target, epigenetic

## Abstract

Glioblastoma (GB), the most aggressive malignant glioma, is made up of a large percentage of glioma-associated microglia/macrophages (GAM), suggesting that immune cells play an important role in the pathophysiology of GB. Under physiological conditions, microglia, the phagocytes of the central nervous system (CNS), are involved in various processes such as neurogenesis or axonal growth, and the progression of different conditions such as Alzheimer’s disease. Through immunohistochemical studies, markers that enhance GB invasiveness have been shown to be expressed in the peritumoral area of ​​the brain, such as Transforming Growth Factor α (TGF-α), Stromal Sell-Derived Factor 1 (SDF1/CXCL12), Sphingosine-1-Phosphate (S1P) and Neurotrophic Factor Derived from the Glial cell line (GDNF), contributing to the increase in tumor mass. Similarly, it has also been described 17 biomarkers that are present in hypoxic periarteriolar HSC niches in bone marrow and in hypoxic periarteriolar GSC niches in glioblastoma. Interestingly, microglia plays an important role in the microenvironment that supports GB progression, being one of the most important focal points in the study of therapeutic targets for the development of new drugs. In this review, we describe the altered signaling pathways in microglia in the context of GB. We also show how microglia interact with glioblastoma cells and the epigenetic mechanisms involved. Regarding the interactions between microglia and neurogenic niches, some authors indicate that glioblastoma stem cells (GSC) are similar to neural stem cells (NSC), common stem cells in the subventricular zone (SVZ), suggesting that this could be the origin of GB. Understanding the similarities between SVZ and the tumor microenvironment could be important to clarify some mechanisms involved in GB malignancy and to support the discovering of new therapeutic targets for the development of more effective glioblastoma treatments.

## Introduction

Among primary brain tumors, glioblastoma (GB) has been described as the most aggressive and is generally associated with a poor prognosis (1). GB is commonly treated with a combination of elements, starting with surgery and followed by radio- and chemo-therapy (2). However, the life expectancy of patients is reduced to approximately 15 months, and they face a high likelihood of the cancer recurring (3). New approaches for the treatment of newly diagnosed and recurrent GB such as Tumor Treating Fields (TTF), have shown a prolonged survival in these patients up to 20 months (4). In addition, GB therapies based on engineering Chimeric Antigen Receptors (CARs) have emerged as an immunotherapeutic approach with high specificity for target tumorigenic cells, but with some adverse effects that must be well defined, in order to design effective control strategies (5).

GB is classified as a grade IV glioma due to its patterns of histological necrosis and vascular changes (6). GB is composed of different types of cells, including glioblastoma stem cells (GSCs) that are responsible for tumor malignancy and expansion (7). Other types of cells that are also present in the tumor mass include NK cells, plasma cells, B cells, gamma delta (γδ) T cells, regulatory T cells (Treg), Follicular helper T (Tfh) cells, Th1, Th17, Th2, naïve CD8+ T cells, EMRA CD8+ T cells, effector memory CD8+ T cells, central memory CD8+ T cells, plasmacytoid dendritic cells, granulocytes, dendritic cells, monocytic cells, macrophages type 2 and type 1, which are the most common cells in GB (8–11). Hira et al. demonstrated that GSC niches are located close to tunica adventitia of a small subset of arterioles in hypoxic areas in GB. Thus, the hypoxic condition of GSH niches promotes the conservation of stem cells (12).

Microglia cells are the resident macrophages in the central nervous system (CNS) and could respond to tumorigenesis signaling by producing chemokines and cytokines that favor tumor progression (1, 13, 14). Glioma-associated microglia/macrophages (GAMs) are abundant in the tumor mass and favor tumor progression (15–17). In the tumor, microglia cells can polarize into two different phenotypes, the typical M1 and M2 phenotype (18). The M1 phenotype is functionally distinguished by its ability to eliminate microorganisms or tumor cells, and to secrete proinflammatory cytokines, such as IL-23, IL-12, IL-6, IL-1β, tumor necrosis factor α (TNF-α), with production of reactive oxygen species (ROS), and a low expression of IL-10 favoring the polarization of T helper cells to Th1 lymphocytes (19, 20); while M2 phenotype is characterized by a low expression of MHC-II, IL-12, IL-23 and a high expression of arginase 1 (Arg1) and anti-inflammatory cytokines, such as TGF-β and IL-10. Thus, M2 phenotype is associated with prolonged neural survival, restriction of brain damage, and prevention of destructive immune responses (21, 22). It has been shown that human GB has a heterogeneous population of M1/M2 macrophages, and M1:M2 ratio is associated with a better prognosis in IDH1 R132H wild-type GB (23). Using automated quantitative immunofluorescence Sørensen et al. found that M2-like TAMs (Tumor associated macrophages) show worse progression in high-grade gliomas and these favor a pro-tumorigenic microenvironment (24). This negative correlation was corroborated by Caponegro et al. (25) and Zhou et al. (26).

Nowadays researchers are focused on discovering the underlying mechanisms of this awful disease, understanding its biology, and researching therapeutic targets to alleviate the symptoms associated with GB, one of which could involve microglial cells’ interactions within the tumor origin and the epigenetics associated therewith.

## Neural Stem Cells, Microglia, and Glioblastoma Stem Cells: Interactions in The Neurogenic Niche

**Figure 1:**
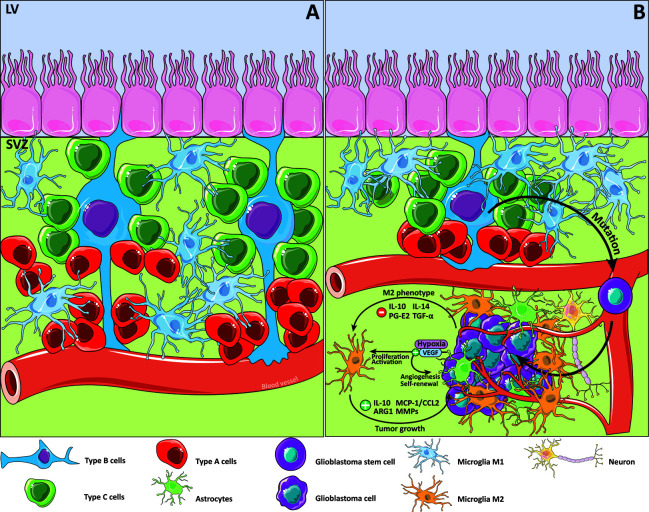
Glioblastoma and the subventricular zone. Details of the subventricular zone (SVZ), microglia cells and its relationship with glioblastoma (GB). Type B cells, knowing as the resident neural stem cells (NSC) within the SVZ are postulated as an origin of glioblastoma stem cells (GSC) because of the accumulation of some mutations. Signaling pathways are modified in response of the changing cancer niche soluble factors that promote M2 microglial cells phenotype. **(A)** SVZ niche in physiological conditions. **(B)** SVZ niche in the GB context.

Neurogenesis is the action through which the neurons are generated out of neural stem cells (NSCs). This process occurs during the embryonic stage and during adulthood where neurogenesis is relegated to two principal regions in the mammalian brain. These specific neurogenic sites are the dentate gyrus of the hippocampus (DG) and the subventricular zone (SVZ) (27, 28). However, other regions have also been described as neurogenic niches, such as the hypothalamus or the striatum in some species (29–31). The SVZ in the lateral ventricles is a neuroepithelium that contains the specific conditions to form and maintain NSCs. NSCs could be differentiated into neurons or glial cells, such as astrocytes, oligodendrocytes, and neurons (**Figure 1A**), and share some specific characteristics with astrocytes (32). NSCs, also called type B cells, embed apical processes into the cerebrospinal fluid, and at the opposite side, embed their basal processes into blood vessels, creating a unique site to drive cell fate according to environmental signaling (33). NSCs are closely related to microglial cells within the SVZ as they are the primary macrophages of the CNS (34). In fact, microglia within the SVZ show a specific morphology, differential expression of some types of receptors, as well as some differences in expression of typical microglial markers such as Iba1, which is underexpressed (35, 36). Furthermore, some studies revealed that microglia release several factors that stimulate migration (37), promote the generation of neuroblasts (38), and enhance not only neurogenesis but also oligodendrogenesis (39). In fact, microglial cells are related to synaptic connectivity, programmed cell death, and regulation of neuronal activity (40–42). All things considered; microglia are a crucial component for determination of NSC fate.

For many years, researchers focused their attention on the SVZ as a potential contributor to GB development. That is because 50%–60% of GB is related to the SVZ and is also associated with the short life expectancy typical of glioblastoma patients (43, 44). This relationship is likely to cause a multifocal diagnosis, as well as an NSC transformation to a new form of cancer cell called glioblastoma stem cells (GSCs) (45). In 2018, Lee et al. described the relationship between GB and its SVZ origin, directing their attention to GSC characteristics using single-cell sequencing to show that some mutations could transform healthy cells into cancer cells (46) (**Figure 1B**). GSCs show similarities with NSCs, such as the capacity to form cell aggregates called neurospheres (47–49), and the expression of several markers such as nestin, Sox2, Musashi-1, or BMI1 (50, 51). It has been postulated that GSCs are responsible for resistance to medical treatments and chemotherapeutic agents such as temozolomide (TMZ) (52–55). GSCs are self-renewing and are important for other components in the tumor origin, such as the microglial cells. Glioma tissues suppress the secretion of some factors, such as TGF-α, IL-10, prostaglandin E2 and IL-14, that promote M2-like microglial phenotype polarization (56), which is implicated in some immune response downregulation processes (57). In tumor masses, M2 microglia are associated with protumorigenic activities that are capable of stimulating tumor growth through several cytokines and chemokines like IL-10, monocyte chemotactic protein-1 (MCP-1/CCL2), some metalloproteinases (MMPs), and ARG1 (13, 42, 58, 59). These factors could affect cell behavior by enhancing the crosstalk between microglia and astrocytes.

Another point associated with the tumor origin is related to the hypoxic environment. In this respect, the Vascular Endothelial Growth Factor (VEGF) is important because it induces the proliferation and activation of microglia and the neural precursor cells are involved in its secretion (60–62). Regarding GB, hypoxia induces angiogenesis and promotes GSCs self-renewal *via* VEGF secretion (63, 64).

Therefore, researchers are now focused on identifying alterations in the signaling pathways and looking for new therapeutic targets to treat GB, focusing on microglia and their relationship with the neurogenic niche.

## Altered Signaling Pathways in Microglia in Glioblastoma

Various alterations have been described in GB signaling pathways that involved microglia (1). Walentynowicz et al. characterized the functional response and transcriptional activity in human and mouse microglial cultures with fresh human cell glioma–conditioned substrate. They found activated pathways related to immune evasion and TGF-β signaling (65). Brennan et al. performed a protein analysis in surgical glioma specimens to identify differential patterns of coordinated switch on between glioma-relevant signal transduction pathways, which revealed three patterns of protein expression and activation: Epidermal Growth Factor Receptor (EGFR) expression related to receptor mutation and amplification; stimulation of the platelet-derived growth factor (PDGF) pathway that is mediated by ligands; or loss of Neurofibromatosis type I NF1 gene expression (66). In addition other researches have shown that the polarized M2 microglia induces the transcription of PDGF Receptor Beta in glioma cells and stimulates their motility capacity (67).

Furthermore, several alterations have also been described in signaling pathway of CCL2 chemokine receptor CCR2 and its major (CCL2/CCR2) GB (68). CCL2 is over-expressed in GB. Interestingly, the secretion level of this chemokine correlates with tumor grade. Glioma cells initially secrete low levels of CCL2 to chemotactically attract microglia cells, which increase CCL2 generation in the tumor environment. The amplified secretion of CCL2 by microglial cells recruits even more microglial cells into the tumor, stimulating the progression and development of the glioma (69).

Relevant findings from Hira et al. show that Mesenchymal Stem Cells (MSCs), expressing SDF-1α and OPN, capture CXCR4-CD44 positive GSCs into GSC niches and protect them from chemotherapy and irradiation (12).

Another altered signaling pathway associated with a negative regulation of T-cells, promoted by microglia, is the Programmed cell Death protein 1 (PD-1), due to the overexpression of ligand Programmed cell Death-Ligand 1 (PD-L1), in GB cells. The PD1 pathway alteration increases the possibility of PD-1/PD-L1 binding in microglia, which is associated with an increased invasion of GB cells into the brain tissue (70).

In addition, blocking the myeloid checkpoint of Signal regulatory protein alpha (SIRPα)/CD47 has shown to be efficient improving tumor phagocytosis and thus decreasing tumor burden (71, 72). SIRP-α in microglia exerts action in the neuronal CD47 to repress microglial stimulation (73). SIRP-α has a receptor tyrosine-based inhibitory motif (ITIM) in its cytoplasmic region (74) that is phosphorylated after CD47–SIRP-α interaction, promoting the binding and activation of SHP-1 and SHP-2 [(Src 2 (SH2) -like domain possessing protein tyrosine phosphatases (PTP)],which inhibits phagocytosis by preventing myosin IIA deposition at the phagocytic synapse (75, 76). Hence, the documented overexpression of CD47 in GB tumor cells (71) favors the immunosuppressive characteristics of microglia in the tumor microenvironment.

## Epigenetic Mechanisms in Microglia in The Context of Glioblastoma

The phenotype of microglia is characterized by its own expressed gene pattern. This transcriptional signature is modified when cells are stimulated by a signal, or under pathological conditions such as GB. In this context, under homeostatic conditions, microglia have a transcriptional spectrum of expression with a main signature consisting of *P2RY13*, *TMEM119*, *CX3CR1*, *P2RY12*,*CSF1R*, *MARCKS*, and *SELPLG* genes and a diminished expression of MHC class II and lipid metabolism genes (2, 3). In the context of GB, microglia present higher expression of proinflammatory and metabolic genes, including *SPP1*, *HLA-DR*, *TREM2*, *APOE*, *CD163*, *GPR56*, and several type I interferon genes, which is substantially different from the genetic expression in homeostatic microglia (4). These changes in expression are modulated by epigenetic mechanisms that regulate the accessibility of genetic loci to transcriptional machinery, gene expression levels, and chromatin architecture without altering the sequences in the DNA (5). This can be demonstrated by treatment with Valproic acid, which inhibits class I HDAC catalysis, promotes proteasomal hydrolysis of HDAC2 and primary adult human microglia, and decreases phagocytosis and levels of PU.1 and CD45, indicating that the regulation of the phagocytic activity of the microglia is carried out by epigenetic mechanisms (6). These HDAC inhibitors (HDACi) have a proapoptotic effect on cancer cells, which involves the interruption of the mitochondrial membrane potential and the increasing of acetylation in the protein histone H3 (7).

Global hypomethylation has been reported in 80% of GB (8), showing intratumoral DNA methylation heterogeneity (9). DNA methylation is closely related to the response to temozolomide (TMZ) treatment, with O6-methylguanine-DNA methyltransferase (MGMT) being the only predictive biomarker for a patient’s response to first-line chemotherapy with TMZ (10). Hypermethylated CpG in the promoter of the connexin 30 (*Cx30*) gene have also been establish in grade III and IV GB, but not in grade I and II gliomas. This hypermethylated region is related to Sp1 and Ap2 expression factor recognition sites and it is correlated with progressive downregulation of *Cx30* mRNA and with the degree of GB (11).

**Figure 2:**
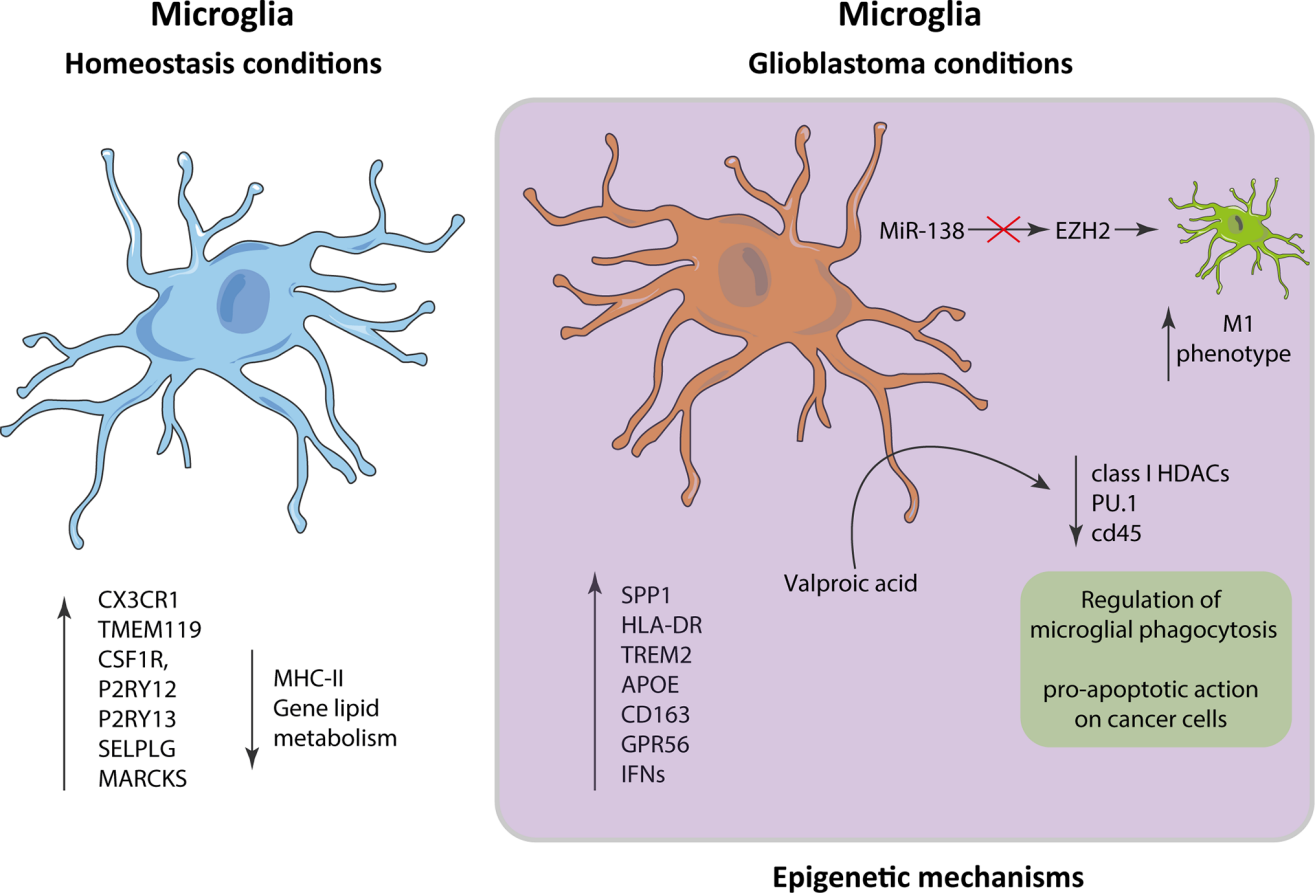
Alterations in microglia in the context of glioblastoma. The gene expression patterns in microglia in homeostatic conditions vs glioblastoma differ significantly, presenting in the latter an inflammatory pattern characterized by an increase in the expression of *SPP1*, *HLA-DR*, *TREM2*, *APOE*, *CD163*, *GPR56*, and interferons.

MiR-138 has been found to effectively inhibit cell division in GB *in vitro* and tumorigenicity *in vivo* by arresting a transcription factor EZH2–mediated signaling loop (12). Inhibition of EZH2 in GB decreases the transcription of M2 profile and increases the expression of M1 related proteins in microglia cells (13). We still have a long way to understand the role that epigenetic modifications play in microglia in GB, but research effort is focused on bringing light to this issue (**Figure 2**).

A detailed view of gene expression of microglia under homeostatic conditions versus GB, supports the understanding of the dysregulation processes in this disease, and could help to find new GB therapeutic targets.

## Microglia as a Therapeutic Target for Glioblastoma

Tumor-associated microglia have been shown to be a key therapeutic target in GB (14) since microglia cells decline in animal experimental models reduces tumor growth (15). Thus, therapies based on microglia as a target could complement the treatments currently used against this disease. Among the molecules that block microglial/macrophages’ infiltration of GSC-derived tumors, the integrin inhibitor arginine-glycine-aspartic acid (RGD) peptides have been shown to interfere with GSC-secreted periostin, thereby these peptides could suppress tumor growth and augment survival of GB-bearing animals (16).

Another strategy employed promotes the antitumor activities of GAMs. For example, in an experimental model of GSC tumors derived from humans implanted in non-obese mice with combined diabetic/severe immunodeficiency (NOD-SCID), it was shown that systemic administration of amphotericin B (AmpB) significantly reduces tumor growth and increases the chances of survival. In animals treated with AmpB, a greater tumor penetration of M1 macrophages and microglial cells was found (77), showing a significant positive regulation of iNOS, and resulting in a higher production of cytotoxic nitric oxide (NO). In these same experiments, positive effects of AmpB it was also found in immunocompetent C57BL/6 mice against very aggressive tumors from stem-enriched CD133 + GL261 glioma cells (17).

Additionally, mTORC plays a key role in the integration of c-MET and PDGFRα signal transduction that are co-activated with EGFR in the context of GB, and it has been shown that inhibition of mTOR activity in rat microglial cells can promote its antitumor properties while restricting its protumorigenic characteristics. Therefore, mTOR inhibitors have the potential to attack both glioblastoma and the protumor functions of GAMs (78–81).

Intracranial injection of a viral recombinant adeno-associated vector (rAAV2) expressing IL-12, induce an increased level of IL-12 in tumor-bearing animals, contributing to microglial penetration in the tumor and reactivation of GAMs’ protective effects. This immunological reactivation of GAMs significantly decreases tumor growth and prolongs animal life (19).

An oncolytic virotherapy using Herpes simplex virus type 1 (HSV-1) has been authorized by the FDA for cancer therapy after the optimum completion of clinical trials (20). In GB, the antitumor efficacy of oncolytic HSV-1 (oHSV-1) is determined, in part, by the amount of microglia/macrophages that phagocytize viruses with the ability to express reporter genes. Thus, viral replication was inhibited, forming an unpermissive OV barrier, and avoiding the spread of oHSV-1 in the glioma mass. The decrease in viral replication, in microglial cells, was related to the suppression of some viral genes by phosphorylation of STAT1/3, responsible for suppressing oHSV-1 replication in microglia/macrophages (21). Together, these strategies employ microglia as a promising therapeutic target in treating glioblastoma.

Microglia are executors of the innate immune response and are specialized in sensing and eliminating abnormal cells, however these cells can change their phenotype and become tumor-promoting cells due to the influence of tumor signals. As part of the tumor mass, tumor-associated macrophages (TAM) are interesting therapeutic target based on data that have shown that the antiphagocytic protein CD47 is increased on the surface of cancer cells, allowing them to evade the innate immune system To avoid the interaction of CD47 with SIRP-α, it is used an anti-CD47 monoclonal antibody (mAb). In microglia cells, anti-CD47 could prevent the expression of their protumorigenic phenotype and turn them into a potential weapon, to arrest GB progression (72).

Stupp et al. In a study with 695 patients with glioblastoma who have completed their initial radio-chemotherapy, the combination of tumor treatment fields (TTFields) with maintenance chemotherapy using alkylating agent TMZ demonstrated a statistically significant improvement with a median overall survival of 20.9 months in this group vs. 16 months in the temozolomide-only group (HR, 0.63, 95% CI, 0.53–0.76, P <0.001) (4).

Pang et al. demonstrated the ability of macrophages as cell carriers of drugs. Culture of RAW264.7 cells in presence of LPS and IFN-γ, shown that these molecules bind to Toll-like receptor 4 and the IFN-γ receptor respectively, activating and promoting the exocytosis of the drug loaded by these cells. Thus, they propose the use of patient-derived M1-type macrophages loaded *in vitro* with the drug of interest, and then transferring them back to the patient to treat GB (82).

## Conclusion

Microglia and TAMs comprise up to 30% of cells in the brain tumor environment (56, 83–88). Microglia cells in the CNS are keys regulators of homeostasis, but their function in immunological surveillance of glioma cells remains little known. Tumor cells, through the expression of different surface and secreted molecules, modulate the phagocytic activity of microglia by altering various signaling pathways and epigenetic mechanisms. Therefore, the modulation and reeducation of the set of microglia constitute a promising antitumor strategy against glioblastoma.

## Author Contributions

NG-D and EN conceptualized the study. IS-G and CF contributed to the methodology. LG and IS-G conducted the formal analysis. RN conducted the investigation. EN provided the resources. NG-D and EN was in charge of the data curation. RN, CF, NG-D, and EN wrote and prepared the original draft. RN, AS, CF, LG, EP, IS-G, NG-D, and EN wrote, reviewed, and edited the manuscript. IS-G and NG-D created the cartoon in **Figures 1** and **2**. All authors contributed to the article and approved the submitted version.

## Funding

We declare that the funds or sources of support received in this specific internal report study were from the Univesidad Simón Bolívar, Clinica de la Costa, Colombia, and Universidad de Cádiz, España, and that the external funding was from the Ministry of Science, Technology And Innovation of Colombia—COLCIENCIAS, subsidy 125380763038 and 125380763188 to EQ. We clarified that the funder had no role in the design of the study, in the collection and analysis of data, in the decision to publish, or in the preparation of the manuscript.

## Conflict of Interest

Authors LG and EN were employed by company Care4You.

The remaining authors declare that the research was conducted in the absence of any commercial or financial relationships that could be construed as a potential conflict of interest.
